# Benefit and Harm of Active Surveillance for Biopsy-proven Renal Oncocytoma: A Systematic Review and Pooled Analysis

**DOI:** 10.1016/j.euros.2022.04.009

**Published:** 2022-05-19

**Authors:** Michael Baboudjian, Daniel Moser, Takafumi Yanagisawa, Bastien Gondran-Tellier, Eva M. Compérat, Damien Ambrosetti, Laurent Daniel, Cyrille Bastide, Shahrokh F. Shariat, Eric Lechevallier, Pietro Diana, Alberto Breda, Benjamin Pradere, Romain Boissier

**Affiliations:** a Department of Urology and Kidney Transplantation, Aix-Marseille University, APHM, Conception University Hospital, Marseille, France; b Department of Urology, Aix-Marseille University, APHM, North University Hospital, Marseille, France; c Department of Urology, Fundació Puigvert, Autonoma University of Barcelona, Barcelona, Spain; d Department of Urology, Comprehensive Cancer Center, Medical University of Vienna, Vienna, Austria; e Department of Urology, The Jikei University School of Medicine, Tokyo, Japan; f Department of Pathology, Medical University of Vienna, Vienna, Austria; g Department of Pathology, CHU Nice, University of Côte d'Azur, Nice, France; h Department of Pathology, Aix-Marseille University, APHM, La Timone University Hospital, Marseille, France

**Keywords:** Oncocytoma, Renal, Biopsy, Surveillance, Review

## Abstract

**Context:**

Active surveillance (AS) of biopsy-proven renal oncocytomas may reduce overtreatment. However, on biopsy, the risk of misdiagnosis owing principally to entities with peculiar hybrids and overlap morphology, and phenotypes argues for early intervention.

**Objective:**

To assess the benefit and harm of AS in biopsy-proven renal oncocytoma.

**Evidence acquisition:**

A systematic review was conducted according to the Preferred Reporting Items for Systematic Reviews and Meta-analyses (PRISMA). We systematically searched PubMed, Scopus, and Web of Science databases from September 26 up to October 2021, for studies that analyzed the outcomes of AS in patients with biopsy-proven renal oncocytoma.

**Evidence synthesis:**

A total of ten studies with 633 patients met our inclusion criteria and were included for analysis. After a median follow-up of 34.5 mo (95% confidence interval [CI] 30.6–38.4), the overall definitive treatment rate from AS to definitive treatment was 17.3% (*n* = 75/433, six studies). The pooled pathological agreement between the initial renal mass biopsy and the surgical pathology report was 91.1%. The main indications for surgery during follow-up were rapid tumor growth and patient request. The pooled median growth rate was 1.55 mm/yr (95% CI 0.9–2.2). No metastasis or death related to renal oncocytoma was reported.

**Conclusions:**

Annual tumor growth of biopsy-proven renal oncocytoma is low. AS is oncologically safe, with favorable compliance of patients. Crossover to definitive treatment revealed a strong concordance between biopsy and final pathology. Further studies on the long-term outcomes of AS are needed.

**Patient summary:**

In this study, we examined the benefit and harm of active surveillance (AS) in biopsy-proven oncocytoma. Based on the available data, AS appears oncologically safe and may represent a promising alternative to immediate treatment. Patients should be included in AS decision discussions.

## Take Home Message

The median growth rate of biopsy-proven renal oncocytoma is 1.55 mm/yr. The pathological agreement between the initial renal mass biopsy and the surgical pathology report is 91.1%. No metastasis or death during active surveillance of renal oncocytoma has been reported.

## 1 Introduction

Renal oncocytomas are benign tumors that account for 3–7% of all solid renal masses but represent up to 18% of small renal masses (SRMs) [1]. The differentiation of renal oncocytoma from renal cell carcinoma (RCC) such as chromophobe RCC is challenging, and imaging characteristics alone are unreliable [2]. For many years, the diagnosis of renal oncocytomas was based on postoperative pathological analysis owing to the low rate of preoperative biopsy in the management of SRMs [3].

The incidental diagnosis of SRMs has increased in the past few decades [4] with a consequential increase in benign tumor incidence, which has been reported in up to 30.9% of the cases [5]. To avoid overtreatment of SRMs, renal mass biopsies (RMBs) have gained interest in this setting [6]. There is growing evidence that RMBs have good accuracy for the diagnosis of SRMs [7] and that a routine RMB is associated with a reduction in unnecessary surgical procedures for benign tumors [6].

In order to reduce the overtreatment of renal oncocytomas [3], active surveillance (AS) for biopsy-proven renal oncocytoma has been proposed. However, the lack of reliability of RMBs to distinguish renal oncocytoma from other tumors included in the oncocytic neoplasm spectrum such as chromophobe RCC and the risk of missing other oncocytic tumors in an AS setting remain controversial [8]. To improve decision-making for the management of renal oncocytoma, we conducted a systematic review and pooled analysis of the benefit and harm of AS in biopsy-proven renal oncocytoma.

## 2 Evidence acquisition

### 2.1 Protocol and registration

We conducted a systematic review in line with the Preferred Reporting Items for Systematic Reviews and Meta-analyses (PRISMA) guidelines [9]. A protocol was submitted to PROSPERO (registration number: CRD42021281340).

### 2.2 Search strategy

A literature search was conducted up to October 2021 in PubMed/Medline, Scopus, and Web of Science databases. Studies were included if they comprised patients with biopsy-proven oncocytoma (patients) managed with AS (intervention) to assess the benefit and harm (outcomes). There was no comparator in this study, and all study designs were included. The following keywords were used in our search strategy: (oncocytoma OR oncocytic) AND (biopsy OR surveillance) AND (kidney OR renal). Initial screening was performed independently by two investigators based on the titles and abstracts of articles to identify ineligible reports. The reasons for exclusions were noted. Potentially relevant reports were subjected to a full-text review, and the relevance of the reports was confirmed after the data extraction process. Disagreements were resolved by consultation with a third coauthor.

### 2.3 Inclusion and exclusion criteria

We included studies that analyzed patients with biopsy-proven renal oncocytoma managed with AS. In case of duplicate publications, either the higher-quality or the most recent publication was selected. Reviews, meta-analyses, letters, editorials, meeting abstracts, author replies, case reports, and non-English articles were excluded. No restriction on the publication date was applied.

### 2.4 Data extraction and analysis

Two review authors (M.B. and D.M.) performed an independent initial screening based on the titles and abstracts, and noted the causes for exclusion of ineligible reports. Both authors independently extracted the following variables from the included studies: first author’s name, publication year, country of research, study design, period of patient recruitment, number of patients included, baseline median tumor size (mm), median follow-up (mo), AS protocol, tumor growth (mm/yr), conversion to definitive treatment (rate, indications, risk factors, and correlation between RMBs and surgical pathology whether partial nephrectomy [PN] or radical nephrectomy was performed), complications and renal function (between patients who remained under AS and those who underwent definitive treatment), metastasis-free survival, and overall survival. All discrepancies regarding data extraction were resolved by consensus with a third coauthor (R.B.).

### 2.5 Risk of bias assessment

The risk of bias (RoB) of the included studies was evaluated according to the “Risk Of Bias In Non-randomized Studies of Interventions (ROBINS-I)” tool [10]. ROBINS-I is the recommended tool to be used in Cochrane Reviews for nonrandomized studies of interventions. In addition, two reviewers independently assessed the RoB using five confounding factors that were identified a priori: interval between two radiological examinations, type of imaging on diagnosis and follow-up, reason for crossover from AS to definitive treatment, oncocytoma differentiated from other oncocytic tumors, and annual tumor growth. RoB summary and graph figures were generated using the Cochrane Review Manager 5.4 (RevMan 5.4; The Cochrane Centre, Copenhagen, Denmark). The overall RoB level was judged as “low,” “unclear,” or “high” risk.

### 2.6 Statistical analysis

Measures of interest included initial tumor size (mm), annual growth rate (measured in mm/yr), and duration of follow-up (mo). The “metamedian” package in R (R Foundation for Statistical Computing, Vienna, Austria) was applied to estimate the pooled median, as described by McGrath et al [11]. The rates of metastasis and death from any cause were extracted as numbers and proportions from the selected articles. All analyses were performed using R Version 4.0.2 (R Foundation for Statistical Computing).

## 3 Evidence synthesis

### 3.1 Study selection and characteristics

**Fig. 1:**
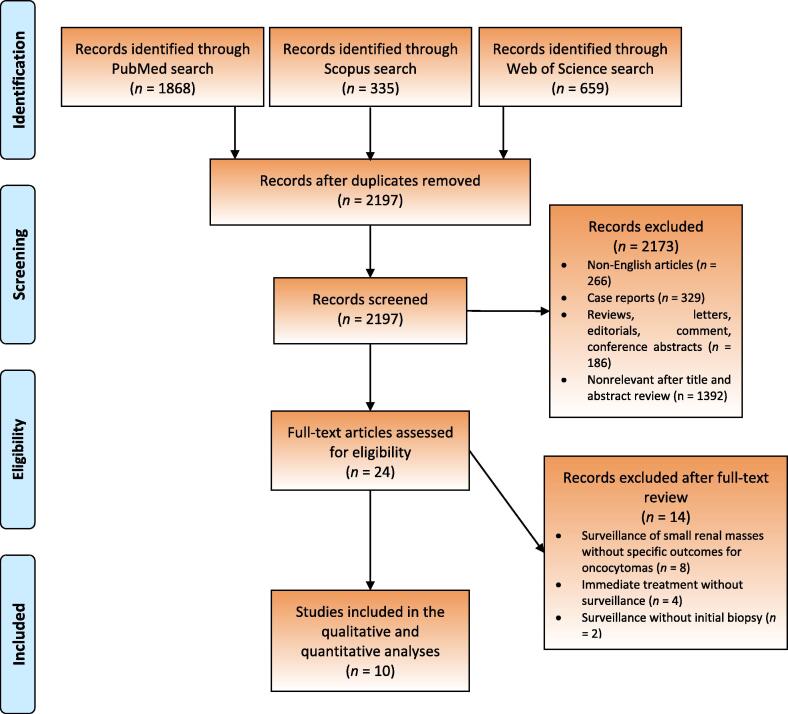
PRISMA flow chart. PRISMA = Preferred Reporting Items for Systematic Reviews and Meta-analyses.

The study selection process is outlined in the PRISMA flow diagram (Fig. 1). A total of 2862 initial searches were identified. After duplicate removal, title and abstract screening, and full-text review, ten studies were included for qualitative and quantitative analyses [12], [13], [14], [15], [16], [17], [18], [19], [20], [21].

The baseline characteristics of the included studies are presented in Table 1. All the studies included (*n* = 10) were retrospective. Of these, six were noncomparative studies, one reported a comparison between AS and PN, and three reported a comparison between oncocytoma and other RCCs. Sample sizes ranged from 15 to 98 cases including a total of 633 patients, baseline tumor size varied from 15 to 34 mm, and median follow-up varied between 29 and 43 mo.

**Table 1:** Overview of the main study characteristics that evaluated active surveillance in biopsy-proven renal oncocytoma

Author	Year	Country of research	Study design	Study period	Number of patients included	Baseline median tumor size (mm)	Median follow-up (mo)
Neuzillet et al [17]	2005	France	Retrospective	1998–2004	15	34	40.1
Kawaguchi et al [20]	2011	Canada	Retrospective	2004–2010	29	26	40
Kurup et al [21]	2012	USA	Retrospective	2000–2009	25	15	33
Richard et al [13]	2016	Canada	Retrospective	2003–2014	79	NA	43
Liu et al [19]	2016	Australia	Retrospective	2000–2014	53	30	34
Alderman et al [14]	2016	USA	Retrospective	2006–2013	96	28	33
Miller et al [15]	2018	USA	Retrospective	2003–2016	78	NA	39.8
Neves et al [18]	2021	UK	Retrospective	2012–2019	98	34	29
Deledalle et al [12]	2021	France	Retrospective	2010–2016	89	26	36
Meagher et al [16]	2021	USA/Italy	Retrospective	2006–2018	71	26	35.3

NA = not available.

### 3.2 RoB in the studies

**Fig. 2:**
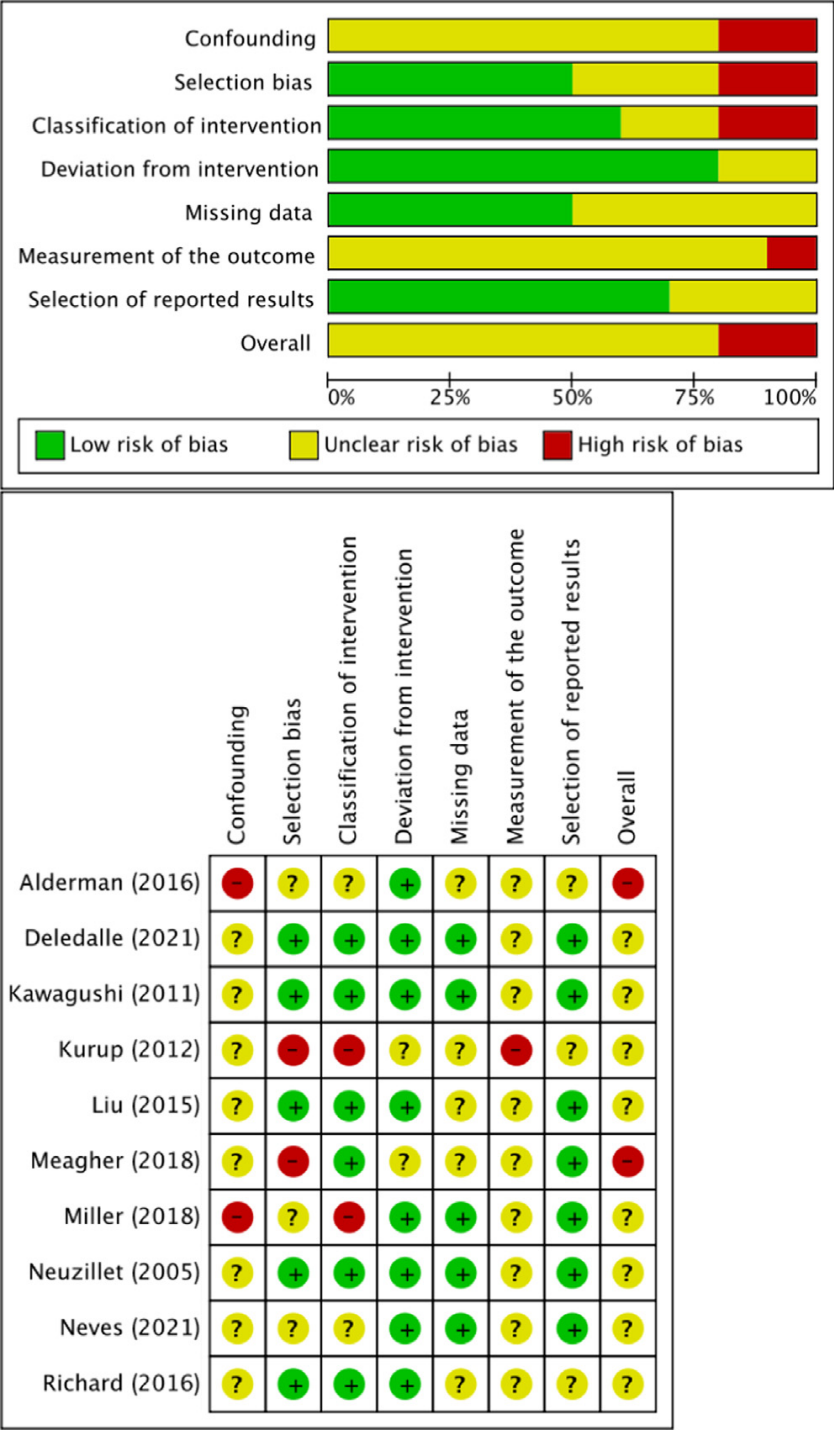
Risk of bias assessment using the ROBINS-I tool. Low risk of bias is indicated by the color green, high risk of bias by red, and some concern by yellow. ROBINS = Risk Of Bias In Non-randomized Studies of Interventions.

**Fig. 3:**
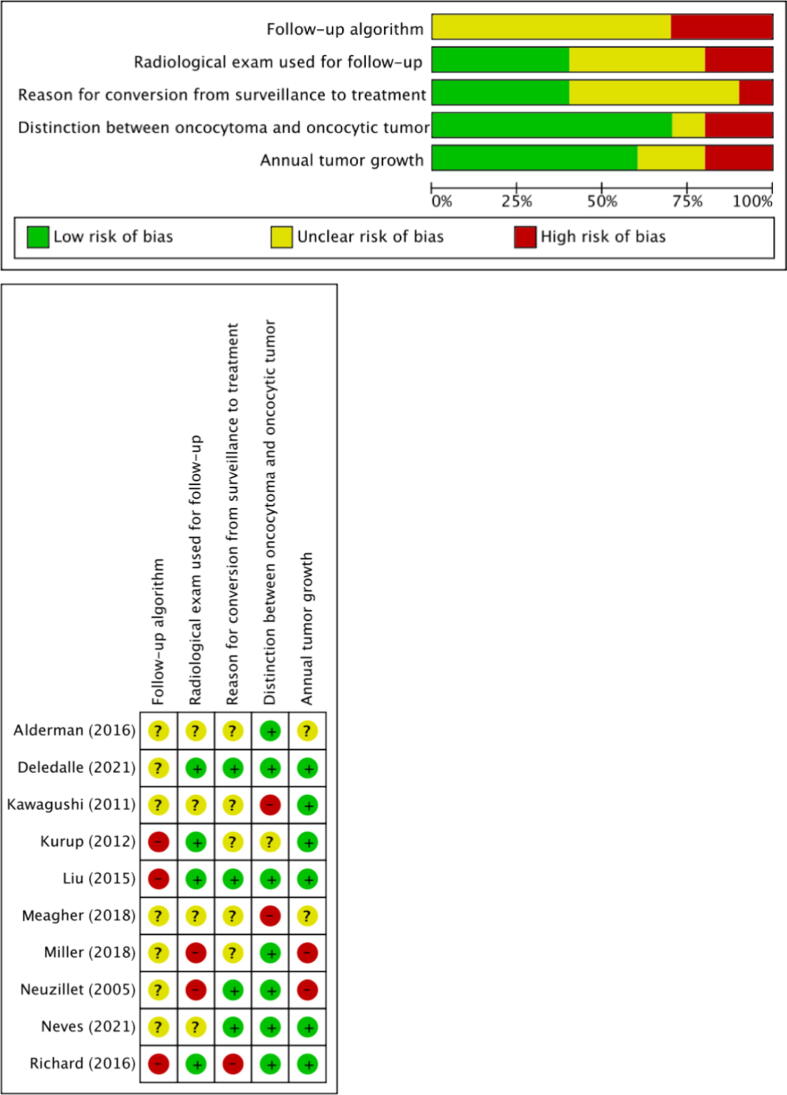
Risk of bias assessment using five confounding factors defined a priori. Low risk of bias is indicated by the color green, high risk of bias by red, and some concern by yellow.

RoB assessments are summarized in Fig. 2 (using ROBINS-I tool) and Fig. 3 (using the five confounding factors defined a priori). All studies were judged to have a moderate to high RoB using the ROBINS-I tool. Similarly, the RoB using the five confounding factors was found to be unclear or high for most domains assessed.

### 3.3 Conversion from AS to definitive treatment

Six studies including 433 patients reported the definitive treatment rates from AS to definitive treatment [12], [13], [14], [17], [18], [19]. In the pooled studies, the median follow-up was 34.5 mo (95% confidence interval [CI] 30.6–38.4). The proportion of patients undergoing a delayed intervention after an initial AS period ranged from 5% to 40%. An analysis of pooled data indicated an overall definitive treatment rate of 17.3% and a definitive treatment rate of 6% per year. Definitive treatments consisted of 35 ablative therapies and 40 surgeries. The types of ablative therapy (radiofrequency or cryoablation) and surgery (PN or radical nephrectomy) used were not reported in all the studies, preventing an accurate comparison of the frequency of definitive treatments used.

Indications for invasive treatment were reported in five studies [12], [13], [17], [18], [19] involving a total of 49/334 patients who underwent definitive treatment (surgical excision or ablative treatment). When reported, indications for definitive treatment were rapid tumor growth (*n* = 32/49, 65%), patient request (*n* = 6/49, 13%), onset of symptoms (*n* = 5/49, 10%), change in tumor shape (*n* = 4/49, 8%), and large tumor volume at baseline (>100 and 87 mm, *n* = 2/49, 4%).

Three studies assessed the risk factors for crossover to definitive treatment [12], [17], [19]. Age on diagnosis was correlated with the likelihood of definitive treatment in every study [12], [17], [19]. Other identified factors were a lower Charlson comorbidity index (4 vs 2; *p* < 0.01), a higher tumor growth rate during follow-up (3.8 vs 1.5 mm/yr; *p* < 0.001) [12], and a larger tumor size at baseline (50.0 vs 27.3 mm, *p* = 0.02) [17].

Five studies compared the pathological correlation between the initial renal mass biopsy and the final specimen [12], [14], [17], [18], [19]. Among the 34 patients included, 31 had a confirmed oncocytoma and two a hybrid tumor, and in one case a low-grade RCC with oncocytic features could not be excluded. Pooled data indicate that the concordance rate between the biopsy and the final specimen pathological examination was 91.1%.

### 3.4 Tumor growth

The annual tumor growth rates were reported in seven studies, including 451 patients [12], [13], [15], [18], [19], [20], [21]. The pooled median initial tumor size was 25.8 mm (95% CI 17–34.6). The pooled median growth rate was 1.55 mm/yr (95% CI 0.9–2.2 mm/yr) with moderate heterogeneity between studies (median growth rates ranged from 1 to 2.7 mm/yr). Growth rate distribution was reported in four studies (265 patients and 276 tumors) [12], [18], [19], [21]. Tumor size decreased in 38 cases (13.8%), a growth rate of <5 mm/yr was reported in 199 tumors (72.1%), and a growth rate of >5 mm/yr was reported in 39 cases (14.1%).

Three studies assessed the factors associated with tumor growth [12], [13], [21]. Two studies failed to identify the predictive factors of growth [12], [21], while Richard et al [13] found that the initial tumor size was associated with tumor growth in a multivariate analysis. However, this analysis was conducted in a cohort of patients with oncocytic neoplasm (oncocytoma and chromophobe RCC) [13].

### 3.5 Renal function in patients under AS versus treated patients

Renal function was assessed in two studies [15], [16]. Meagher et al [16] compared renal function outcomes in patients under AS versus those who underwent PN. A total of 295 patients were analyzed (224 PN/71 AS, median follow-up 37.4 mo). The mean estimated glomerular filtration rate (eGFR) at the last follow-up was lower in the AS group (64.3 vs 70.5 ml/min/1.73 m^2^ in the PN group, *p* = 0.03), with a higher rate of de novo eGFR of <60 ml/min/1.73 m^2^ (28.2% vs 12.1%, *p* < 0.001). In a cohort of renal oncocytic neoplasms, Miller et al [15] compared the decline in renal function at 1 and 3 yr of follow-up with baseline renal function (%), according to the treatment used. Renal function following treatment was lower for patients who underwent radical nephrectomy at 1 (78.5% glomerular filtration rate [GFR]) and 3 (77.4% GFR) yr versus PN (94.2% and 96.6%, respectively), ablation (90.0% and 94.2%, respectively), and AS (99.6% and 95.9%, respectively; *p* < 0.01).

### 3.6 Complications in patients under AS versus treated patients

Meagher et al [16] compared complication rates in patients under AS versus treated patients. The rate of complications observed in the PN group was higher (45/224, 20%) than that in the AS group (7/71, 9.9%), but the difference did not reach statistical significance (*p* = 0.051). High-grade complications were recorded only in the PN group (10/224, 4.5%). In the series of Deledalle et al [12], no complications were reported in patients who remained under AS, while three major complications were recorded (three/24, 13%) in patients who had definitive treatment.

### 3.7 AS Protocol

The AS protocol was described in five studies [12], [13], [17], [19], [21]. Magnetic resonance imaging, ultrasound, and computed tomography examinations were, respectively, used in three [12], [13], [21], four [12], [13], [17], [19], and five [12], [13], [17], [19], [21] protocols. The frequency of radiological examinations varied between studies: every 3–6 mo [13], every 6–12 mo [17], [19], [21], or at least once a year [12].

### 3.8 Oncological outcomes

Five studies assessed metastasis-free survival, including 395 patients [12], [13], [14], [15], [19]. After a pooled median follow-up of 35 (95% CI 31–39) mo, no metastasis was recorded.

Overall survival was reported in two studies [13], [18]. After a pooled median follow-up of 35.4 (range 21.7–49) mo in 177 patients, six deaths (3.4%) unrelated to renal oncocytoma occurred.

### 3.9 Discussion

#### 3.9.1 Main findings and interpretation of the results

In this systematic review and pooled analysis, we found that AS appeared to be a safe therapeutic option for biopsy-proven oncocytoma. With no oncocytoma-related deaths or distant metastases reported, and a pooled median growth rate of 1.55 mm/yr, AS is safe without missing the window of opportunity to perform surgery later during follow-up. Indeed, the pooled definitive treatment rate was only 17.3% among the studies. Nevertheless, our findings underline the lack of actual consensus regarding the criteria to decide for definitive treatment.

In the present review, >85% of renal oncocytomas had slow or no growth. The overall growth rate observed in the pooled studies was 1.55 mm/yr, which is consistent with other reports dealing with AS of SRMs [22], [23]. There are some data comparing growth rates between different pathological features, which suggest that growth rates for RCC and oncocytomas are not distinguishable [24], [25]. However, we found that tumor growth was often related to the indication for definitive treatment in patients with AS for biopsy-proven renal oncocytoma.

Throughout the studies, fast tumor growth was the main indication for definitive treatment. The threshold for defining rapid tumor growth was 5 mm/yr [17], [18], [19], but was unreported or subjective in some studies [12], [13]. The indication for definitive treatment based solely on tumor growth is questionable since oncocytomas appear to grow at the same rate as malignant tumors [22], [26]. Moreover, the pooled data indicate that the concordance rate between biopsy and final specimen was 91.1%, highlighting that a large number of definitive treatments in patients with rapid tumor growth could have been avoided. Nevertheless, Patel et al [8] reported a lower concordance with only 31 of 48 (64.6%) oncocytic neoplasms on RMBs to be oncocytomas on final specimen. The pathological similarity of renal oncocytoma to other oncocytic lesions is often debated as to whether a formal and definitive diagnosis of oncocytoma (with typical features on the biopsy specimen) is preferable after biopsy or whether more general terminology, such as oncocytic neoplasm, should be used.

The second most common reason for switching to definitive treatment was patient choice. While patient choice is a crucial and well-studied issue in RCC [26], [27], no data have been reported in the management of renal oncocytoma. Treatment decisions are complex, especially for patients with incidentally diagnosed oncocytoma. However, the present review showed that AS is oncologically safe. Patients should be aware that pathological examination of renal tumor biopsy could miss other oncocytic renal neoplasms in 9%, but deferred intervention is possible in case of atypical evolution. Therefore, no tumor-related deaths or distant metastases were reported in the included studies.

#### 3.9.2 Limitations of the review and included studies

There are several limitations that should be acknowledged. First, the available data on AS of biopsy-proven renal oncocytomas remain limited, with the inclusion of mainly noncomparative and retrospective studies. Although we observed a pooled median follow-up of 34 mo, long-term outcomes are still lacking, preventing clinicians from informing patients of the long-term safety of AS for decision-making purposes.

All the studies were published over a relatively long time frame (1998–2019), during which the indications for an RMB may have varied considerably. Moreover, the inclusion of patients in an AS protocol was left to the discretion of clinicians.

Patel et al [8] showed in their meta-analysis of 205 biopsies of oncocytic renal masses that the positive predictive value of the diagnosis of oncocytoma on biopsy was 67%. However, when individual studies were considered, the confirmation rate on the final surgical specimen for the diagnosis of oncocytoma varied widely, from 25% to 100% [8]. This great heterogeneity shows the important role of interpretation by pathologists and resources implemented for tumor subtyping, such as immunohistochemistry and genetic tools. A possible limitation is that during the early period of analysis, new oncocytic emerging entities such as low-grade oncocytic tumors were not clearly defined in the literature. Nevertheless, these entities are rare and, as opposed to chromophobe RCC, appear to share a favorable outcome with oncocytoma. In the present review, data regarding pathologist experience and additional explorations used were scarce. Therefore, well-designed large-scale trials with a centralized review of pathological slides are required to confirm the findings of the present study.

#### 3.9.3 Implications for practice and future research

This review confirms that we can systematically propose AS to most of our patients with renal oncocytoma as a safe therapeutic option. For the purposes of comparison between AS and immediate treatment, future investigations should also provide medicoeconomic evaluation and patient-reported quality of life.

Although a routine RMB has the potential to reduce surgery for benign tumors, it has been shown that even centers that routinely perform RMBs still miss benign tumors that are operated [7]. The main risk factor reported for crossover to definitive treatment was young age on diagnosis [12], [17], [19]. Definitive treatment for renal oncocytomas may still be indicated in young patients with long life expectancies who do not wish to undergo life-long and stringent surveillance. In any case, surgery should be indicated before missing the window of opportunity to perform a PN in order to limit the risk of renal failure.

Defining standardized criteria for discontinuing AS of renal oncocytomas is mandatory. The threshold of 5 mm/yr to define a rapid growth rate and subsequently indicate definitive treatment is based primarily on low-level evidence [17]. A more relevant biological or radiological threshold could become the standard.

Recently, increasing evidence has shown that technetium-99m (99mTc)-sestamibi [28], [29] and radiomics [30] are promising tools for differentiating renal oncocytomas from clear cell RCCs. Before their clinical implementation, larger studies are necessary to better define the diagnostic accuracy of these images and their exact place in the AS protocol.

## 4 Conclusions

AS of biopsy-proven renal oncocytoma is a safe and feasible alternative to immediate treatment with a cumulative midterm definitive treatment rate of 17.3%. However, the included studies had high RoBs and long-term follow-up data are lacking. To improve decision-making, patient preferences and expectations must be taken into account.

  ***Author contributions*:** Michael Baboudjian had full access to all the data in the study and takes responsibility for the integrity of the data and the accuracy of the data analysis.

*Study concept and design*: Baboudjian, Pradere, Boissier.

*Acquisition of data*: Baboudjian, Moser, Yanagisawa.

*Analysis and interpretation of data*: Baboudjian, Boissier, Gondran-Tellier, Pradere.

*Drafting of the manuscript*: Baboudjian, Pradere, Boissier.

*Critical revision of the manuscript for important intellectual content*: Compérat, Ambrosetti, Daniel, Bastide, Shariat, Lechevallier, Breda, Diana.

*Statistical analysis*: Baboudjian, Gondran-Tellier.

*Obtaining funding*: None.

*Administrative, technical, or material support*: None.

*Supervision*: None.

*Other*: None.

  ***Financial disclosures:*** Michael Baboudjian certifies that all conflicts of interest, including specific financial interests and relationships and affiliations relevant to the subject matter or materials discussed in the manuscript (eg, employment/affiliation, grants or funding, consultancies, honoraria, stock ownership or options, expert testimony, royalties, or patents filed, received, or pending), are the following: None.

  ***Funding/Support and role of the sponsor*:** None.
